# Identification of New HIV-1 Circulating Recombinant Forms CRF81_cpx and CRF99_BF1 in Central Western Brazil and of Unique BF1 Recombinant Forms

**DOI:** 10.3389/fmicb.2019.00097

**Published:** 2019-02-11

**Authors:** Mônica N. G. Reis, Monick L. Guimarães, Gonzalo Bello, Mariane M. A. Stefani

**Affiliations:** ^1^Laboratório de Imunologia da Aids e da Hanseníase, Instituto de Patologia Tropical e Saúde Pública, Universidade Federal de Goiás, Goiânia, Brazil; ^2^Laboratório de Aids e Imunologia Molecular, Instituto Oswaldo Cruz, Fundação Oswaldo Cruz, Rio de Janeiro, Brazil

**Keywords:** HIV-1, CRFs, URFs, molecular epidemiology, Central Western, Brazil

## Abstract

Intersubtype recombinants classified as circulating recombinant forms (CRFs) or unique recombinant forms (URFs) have been shown to play an important role in the complex and dynamic Brazilian HIV/AIDS epidemic. Previous *pol* region studies (2003–2013) in 828 patients from six states from Central Western, Northern and Northeastern Brazil reported variable rates of BF1, F1CB, BC, and CF1 mosaics. In this study HIV-1 subtype diversity BF1, F1CB, BC, and CF1 recombinants in *pol* were analyzed. Full/near-full/partial genome sequences were generated from F1CB and BF1 recombinants. Genomic DNA extracted from whole blood was used in nested-PCR to amplify four overlapping fragments encompassing the full HIV-1 genome. Phylogenetic trees were generated using the neighbor-joining/NJ method (MEGA 6.0). The time of the most recent common ancestor (TMRCA) of F1CB and BF1 clades was estimated using a Bayesian Markov Chain Monte Carlo approach (BEAST v1.8; BEAGLE). Bootscanning was used for recombination analyses (Simplot v3.5.1); separate NJ phylogenetic analysis of fragments confirmed subtypes. The phylogenetic analyses of protease/reverse-transcriptase sequences in 828 patients revealed 76% subtype B (*n* = 629), 6.4% subtype C (*n* = 53), 4.2% subtype F1 (*n* = 35), 13.4% intersubtype recombinants: 10.5% BF1 (*n* = 87), 2.3% BC (*n* = 19), 0.4% F1CB (*n* = 3), and 0.2% CF1 (*n* = 2). Two full and one partial BF1C genomes allowed the characterization of the CRF81_cpx that has 9 breakpoints dividing the genome into 10 subregions. Basic Local Alignment Search Tool searches (Los Alamos HIV Sequence Database) identified six other sequences with the same recombination profile in *pol*, five from Brazil, and one from Italy. The estimated median TMRCA of CRF81_cpx was 1999 (1992–2003). CRF60_BC-like sequences, originally described in Italy, were also found. Two full and one near full-length BF1 genomes led to the characterization of the new CRF99_BF1 that has six recombination breakpoints dividing the genome into seven subregions. Two new URFs BF1, with six recombination breakpoints and seven subregions were also characterized. The description of the first Brazilian BF1C CRF81_cpx and of the new CRF99_BF1 corroborate the important role of CRFs in the HIV/AIDS epidemic throughout Brazil. Our data also highlight the value of HIV-1 full-genome sequence studies in order to fully reveal the complexity of the epidemic in a huge country as Brazil.

## Introduction

Recombination of HIV-1 group M subtypes is considered a driving force for its genetic diversity worldwide (Worobey and Holmes, 1999). Brazil is a vast country with remarkable socio-economic and geographic differences where a very complex and dynamic HIV/AIDS epidemic has been reported. HIV-1 subtype B predominates in all geographic regions, except in Southern region where subtype C is a major variant (Soares et al., 2005; Gräf and Pinto, 2013; Delatorre et al., 2017). The distribution of F1 subtype has also significant regional variances ranging from low prevalence, in most regions, to high frequency in Pernambuco state, Northeastern Brazil (Cavalcanti et al., 2012; Gaspareto et al., 2012; Silveira et al., 2012; Lima et al., 2016a). The cocirculation of distinct HIV-1 subtypes is known to increase the chances of coinfection and of the generation of BF1, F1CB, BC, and CF1 intersubtype recombinant viruses (Jetzt et al., 2000; Faria et al., 2014). In the last decade, different circulating recombinant forms (CRFs) and unique recombinant forms (URFs) have been described including BC recombinants especially in the Southern and BF1 recombinants in different Brazilian regions (Guimarães et al., 2008, 2010; Passaes et al., 2009; Ferreira et al., 2011; da Silveira et al., 2012; Pessôa et al., 2014b, 2016; Moura et al., 2015a,b; Gräf et al., 2016; Lima et al., 2016b; Reis et al., 2017).

Previous studies from our group in the HIV-1 *pol* region reported variable rates of intersubtype recombinant BF1, F1CB, BC, and CF1 viruses among over 800 patients living in six Brazilian States located in the Central Western, Northern, and Northeastern Brazilian regions (Cardoso et al., 2009, 2010, 2011; Cardoso and Stefani, 2009; Carvalho et al., 2011; Ferreira et al., 2011; Alcântara et al., 2012; da Silveira et al., 2012; da Costa et al., 2013; Moura et al., 2015a,b; Lima et al., 2016b). Recently near full and full-length genomes revealed a noteworthy diversity of BF1 recombinants among these patients and allowed the identification of the CRF90_BF1 and of several URFs BF1 (Reis et al., 2017). In this study, we summarize HIV-1 molecular data in *pol* among this large group of patients from six Brazilian states. Additionally, we describe the frequency and profile of intersubtype recombinants. Full and near-full length genome sequences of F1CB and BF1 intersubtype mosaics allowed the characterization of two new CRFs identified in Central Western Brazil and partial genomes characterized two new URFs BF1.

## Materials and Methods

### Study Samples

Our study population included 828 HIV-1 infected individuals recruited in Central Western (Goiás, Mato Grosso, and Mato Grosso do Sul states), Northern (Tocantins state), and Northeastern (Maranhão and Piauí states) Brazilian regions from 2003 to 2013. Patients belonged to the following groups: recently diagnosed antiretroviral/ARV-naïve (701/828; 84.7%), therapeutic failure of ARV treatment (127/828; 15.3%), inmates (27/828; 3.3%), and pregnant women (278/828; 33.6%). These studies were approved by the local Institutional Ethics Committee and all participants signed an informed consent form before blood collection (Goiás: protocols #073/05, #003/2008, #163/2010, at CEPMHA/HC/UFG; Mato Grosso: protocol #435/07 at Universidade Federal do Mato Grosso/UFMT; Mato Grosso do Sul: protocol #1143 at Universidade Federal do Mato Grosso do Sul/UFMS; Piauí: protocol #022/2011 at Universidade Estadual do Piauí/UESPI; Maranhão: protocol #16/2011 at Hospital de Doenças Tropicais Dr Natan Portela).

### POL Gene Sequencing

Plasma RNA extraction, reverse transcription into complementary DNA (cDNA), amplification by nested polymerase chain reaction, sequencing of the protease (PR) and reverse transcriptase (RT) regions in the *pol* gene and phylogenetic analyses were previously described (Cardoso et al., 2009, 2010, 2011; Cardoso and Stefani, 2009; Carvalho et al., 2011; Ferreira et al., 2011; Alcântara et al., 2012; da Silveira et al., 2012; da Costa et al., 2013; Moura et al., 2015a,b; Lima et al., 2016b).

### Full-Length, Near-Full-Length, and Partial HIV-1 Genomes

Genomic DNA was extracted from whole blood samples (QIAamp^®^ DNA Blood Mini Kit/QIAGEN, Qiagen, Hilden, Germany). The double-stranded proviral HIV-1 DNA was amplified by nested-PCR employing Platinum Taq DNA polymerase (Invitrogen, Carlsbad, CA, United States) into four overlapping fragments [(#1 (408–2594), #2 (2253–4830), #3 (4653–7811), and #4 (6954–9625) relative position to HXB2 genome] using HIV-1 specific primers as described in Reis et al. (2017). The amplified DNA fragments were purified (kit QIAquick^®^ PCR Purification Kit/QIAGEN, Qiagen, Hilden, Germany) and sequenced (*Big Dye Terminator Sequencing Kit v. 3.1*, Applied Biosystems, Foster City, CA, United States; ABI Prism 3100 Genetic Analyzer, Applied Biosystems, Foster City, CA, United States).

### Phylogenetic and Recombination Analyses

Sequences from this study were aligned using Clustal X 2.0 implemented in BioEdit 7.2.0 program (Thompson et al., 1994). Reference sequences of HIV-1 group M subtypes (B, C, and F1) were obtained from the Los Alamos HIV Database (http://hiv.lanl.gov, last accessed December 2018). Phylogenetic trees were generated using the neighbor-joining (NJ) method (Nei and Kumar, 2002) under the Kimura two-parameter model (Kimura, 1980) using MEGA 6.0 software (Tamura et al., 2013). Bootstrap values (BP, 1000 replicates) above 80% were considered significant. Recombination analyses were performed in all viral isolates using the bootscanning method implemented in Simplot v3.5.1 software (Lole et al., 1999) with the following parameters: 300 nucleotide (nt) window, 20 nt increments, NJ method under Kimura’s two-parameter correction with 100 bootstrap replicates. Informative site analyses were used to characterize the recombination breakpoints, for this purpose, consensus sequences from Brazilian HIV-1 subtypes B, C, and F1 were generated in the DAMBE program (Xia and Xie, 2001). The fragments of our study sequences assigned to specific HIV-1 subtypes were confirmed by separate NJ phylogenetic analysis as described above.

Representative samples from the HIV-1 BF1 and F1CB Brazilian clusters herein identified were submitted to a Basic Local Alignment Search Tool (BLAST) analysis in order to recover other sequences with high similarity (>95%) and probably with similar recombination profiles. The BLAST analysis was performed using sequences obtained from the Los Alamos HIV Sequence Database (http://hiv.lanl.gov, last accessed December 2018).

### Evolutionary Analyses of F1CB and BF1 Recombinants

The time of the most recent common ancestor (TMRCA) of HIV-1 F1CB and BF1 clades was estimated using a Bayesian Markov Chain Monte Carlo (MCMC) approach implemented in BEAST v1.8 (Drummond et al., 2002; Drummond and Rambaut, 2007) with BEAGLE to improve run-time (Suchard and Rambaut, 2009). Analyses were performed using the GTR+I+G nucleotide substitution model selected by the jModeltest program (Posada, 2008), a flexible Bayesian Skyline coalescent tree prior that does not require strong prior assumptions of demographic history (Drummond et al., 2002) and a relaxed uncorrelated lognormal molecular clock model (Drummond et al., 2006) with an informative uniform prior interval (1.5–2.5 × 10^-3^ nucleotide substitutions per site per year) as the estimated coefficient of rate variation (mean = 0.30; 95% highest probability density/HPD values: 0.0004–0.65) indicated a significant variation of substitution rate among branches. One MCMC chain was run for 1 × 10^7^ generations. Convergence and uncertainty of parameter estimates were assessed by calculating the effective sample size (ESS) and the 95% HPD, respectively, using Tracer v1.6 (Rambaut and Drummond, 2007). The maximum clade credibility (MCC) tree was summarized with TreeAnnotator v1.8 and visualized with FigTree v1.4.0.

## Data Availability

All HIV-1 sequences generated in this study were deposited in the GenBank database (accession numbers MH938677, MH938678, and MH986013-MH986018).

## Results

### Phylogenetic Analyses of *pol* Region in HIV-1-Infected Patients From Six Brazilian States

**FIGURE 1:**
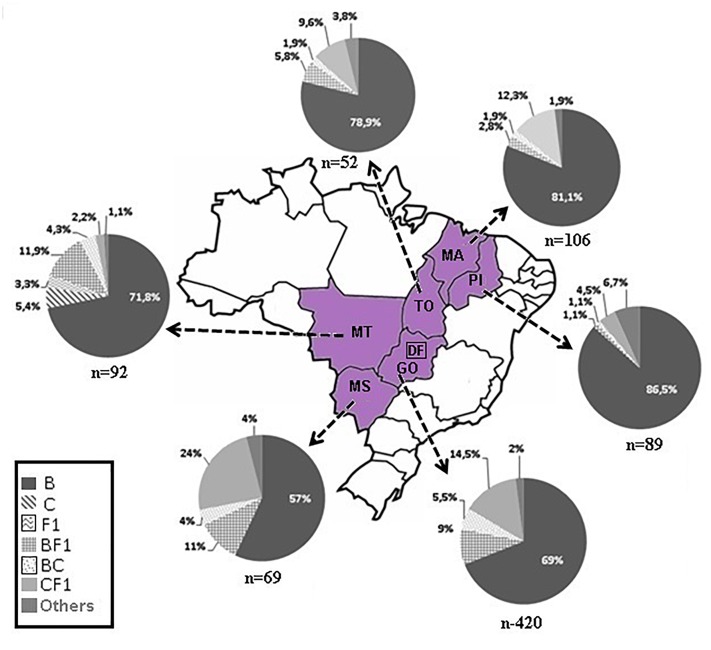
Map of Brazil highlighting our study area. The pie-charts depict the frequency of HIV-1 subtypes of 828 patients living in six states located in three geographic regions, Central Western: Goiás/GO, Mato Grosso/MT, Mato Grosso do Sul/MS; Northern: Tocantins/TO; and Northeastern: Maranhão/MA, Piauí/PI. The number of *pol* sequences analyzed is depicted below each pie chart.

The phylogenetic analyses of the *pol* (PR/RT) region in 828 patients from six Brazilian States showed that 76% was subtype B (629 out of 828), 6.4% was subtype C (53 out of 828), and 4.2% was subtype F1 (35 out of 828). Intersubtype recombinant viruses represented 13.4% (111 out of 828): 10.5% BF1 (87 out of 828), 2.3% BC (19 out of 828), 0.4% F1CB (3 out of 828), and 0.2% CF1 (2 out of 828) (Figure 1; Supplementary Table 1). The BF1 recombinants identified in this dataset were recently described in detail elsewhere (Reis et al., 2017).

### HIV-1 F1CB, BC, and CF1 Recombinant Viruses in the *pol* Region

**FIGURE 2:**
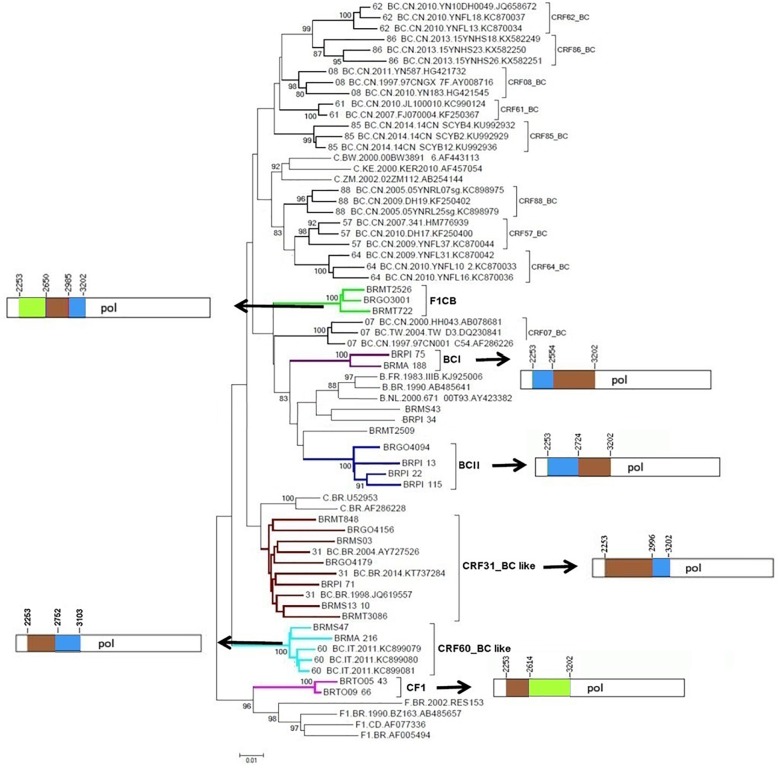
Phylogenetic tree of partial HIV-1 *pol* sequences of F1CB, BC, and CF1 recombinants from Central Western, Northern, and Northeastern Brazil. The tree was constructed using MEGA software, 6.0 version under NJ method and Kimura two-parameter model (Bootstrap value over 80%). The mosaic patterns of recombinants: Cluster F1CB, Clusters BC (I and II), CRF31_BC-like, CRF60_BC-like, and Cluster CF1 are depicted. In the mosaic structure representations of F1CB, BC, and CF1 clusters, the breakpoint positions are indicated according to HXB2 genome position. In the mosaic structure, the green color stands for HIV-1 subtype F1, blue color stands for subtype B, and brown color stands for subtype C.

Phylogenetic and bootscanning analyses of the 23 mosaic sequences composed of subtypes F1CB, subtypes BC, and subtypes CF1 (Supplementary Table 1) showed six clusters with distinct recombination profiles (Figure 2). Three BC recombinant isolates (BRMS43, BRPI34, and BRMT2509) did not cluster with other samples.

The F1CB cluster was composed of three isolates (Goiás = 1, Mato Grosso = 2). The BC-I cluster contained two isolates (Maranhão = 1, Piauí = 1) and the BC-II cluster contained four isolates (Goiás *n* = 1, Piauí *n* = 3). The CRF31_BC-like cluster contained seven isolates (Goiás = 2, Mato Grosso do Sul = 2, Mato Grosso = 2, Piauí = 1). The CRF60_BC-like cluster comprised two isolates (Maranhão = 1, Mato Grosso do Sul = 1) and the CF1 cluster contained two isolates from Tocantins (Figure 2).

### Characterization of the F1CB Cluster in the *pol* Region

The deduced mosaic structures of the three F1CB recombinant strains in *pol* (BRGO3001 from Goiás, BRMT2526 and BRMT722 from Mato Grosso) were compared and the patterns of the shared recombination breakpoints are summarized in Figure 2.

**FIGURE 3:**
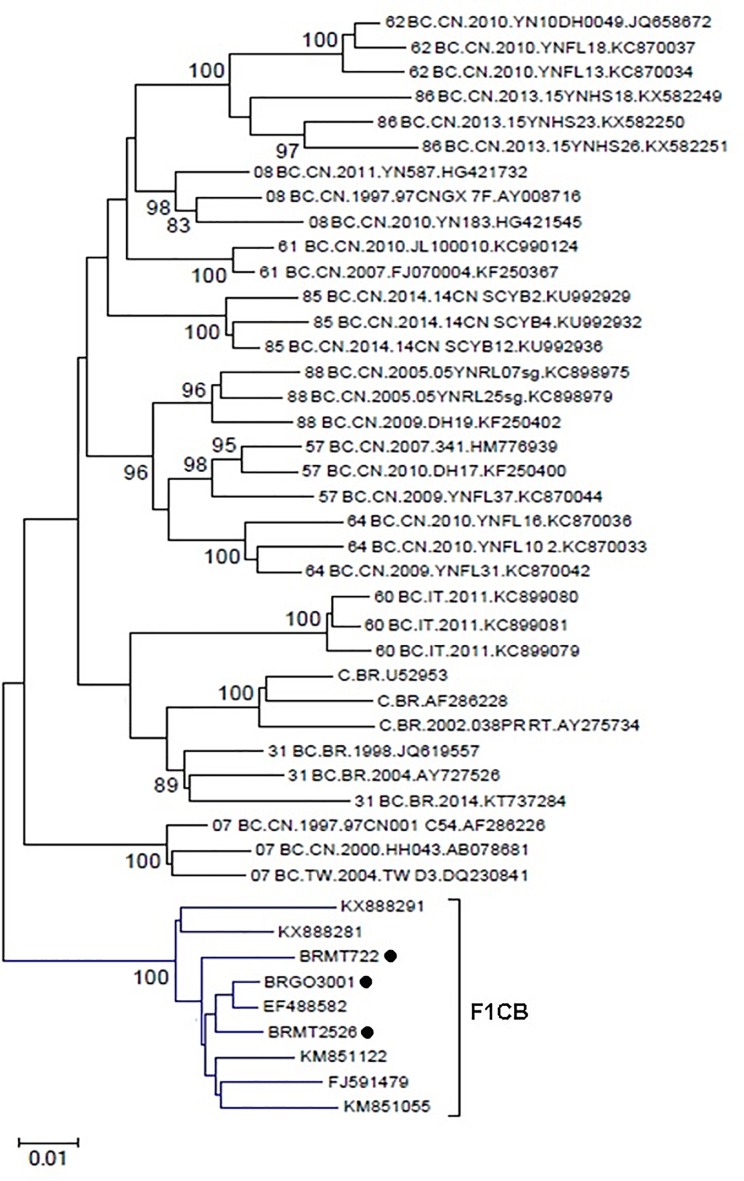
Phylogenetic tree including the three F1CB sequences identified here and the six *pol* sequences sharing over 95% similarity recovered from the BLAST search. The tree was constructed using MEGA software, 6.0 version under NJ method and Kimura two-parameter model (Bootstrap value over 80%).

**FIGURE 4:**
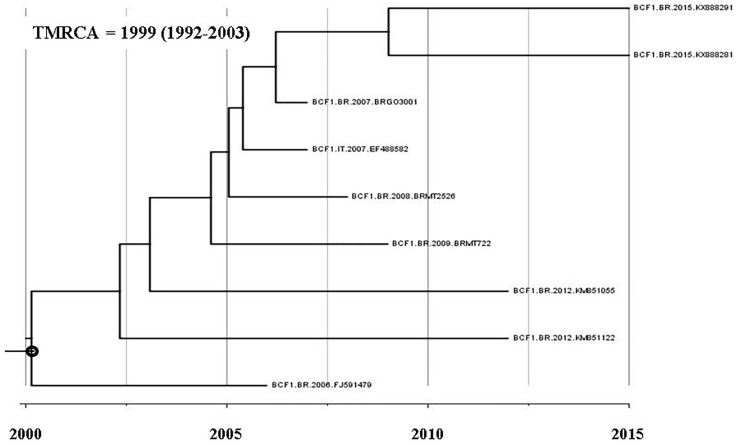
Time-scaled Bayesian MCMC tree of nine *pol* sequences of F1CB HIV-1 sequences.

A BLAST search analysis recovered six other *pol* sequences with high homology with those from F1CB cluster. Five of them were from individuals collected in two Brazilian states (FJ591479, KM851055, and KM851122 from Paraná, Southern region and KX888281 and KX888291 from Mato Grosso). One recovered BF1C *pol* sequence was from Italy. Phylogenetic and recombination analyses of the *pol* F1CB sequences identified here and of these six sequences recovered from the BLAST search showed bootstrap values of 100% and the same mosaic structure pattern (Figure 3). The estimated median TMRCA of the F1CB cluster was 1999 ranging from 1992 to 2003 (Figure 4).

### Description of the CRF81_cpx

Phylogenetic and bootscanning analyses of the near-full and full-length genome sequences from the F1CB cluster: BRGO3001 strain (626–9322 bp), BRMT2526 strain (626–9322 bp), and partial sequences of the BRMT722 strain (626–1677 bp; 2263–4822 bp; 4824–5964 bp; 7855–8653 bp) described here allowed the description of the new CRF81_cpx named according to the standardized nomenclature by the Los Alamos National Laboratory.

**FIGURE 5:**
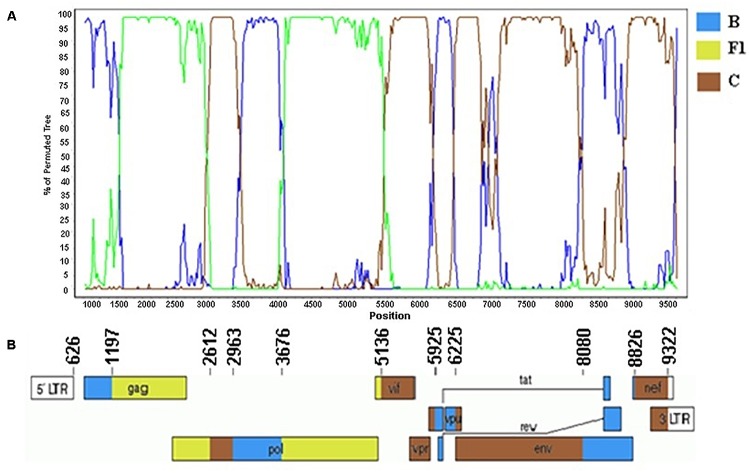
Mosaic structure of the CRF81_cpx composed of HIV-1 subtypes B, F1, and C. **(A)** Bootscanning analysis was conducted using a window size of 300 bp and a step size of 20 bp along with reference strains of representative subtypes B, F1, and C from HIV-1 group M. **(B)** Genomic structure of CRF81_cpx. Breakpoint positions according to HXB2 genome positions are indicated. The blue color stands for HIV-1 subtype B, green color stands for subtype F1, and brown color stands for subtype C. The mosaic map was generated using the Recombinant HIV-1 Drawing Tool (https://www.hiv.lanl.gov/content/sequence/DRAW_CRF/recom_mapper.html).

The genome sequences of the three CRF81_cpx strains showed a complex recombination profile with nine breakpoints, which divide the HIV-1 genome into 10 subregions (Figure 5). The following subregions and subtypes were identified: subtype B (626–1196 bp; 2963–3675 bp; 5925–6224 bp; 8080–8825 bp), subtype F1 (1197–2611 bp; 3676–5135 bp), and subtype C (2612–2962 bp; 5136–5924 bp; 6225–8079 bp; 8826–9322 bp).

The three CRF81_cpx strains were obtained from two 40- and 46-year-old males who lived in Rondonópolis and Cuiabá cites, in Mato Grosso state and from one 33-year-old female living in Alexânia city, in Goiás state. All three patients were ARV-naïve and were blue-collar workers. The 46-year-old patient self-reported to belong to the men who have sex with men (MSM) category and the other two patients were from the heterosexual category.

### Identification of the New CRF99_BF1

We have previously described five highly supported clusters (#1–5) among 87 BF1 recombinants and full-genome sequences from Cluster #5 characterized the new CRF90_BF1 (Reis et al., 2017; Supplementary Table 1).

**FIGURE 6:**
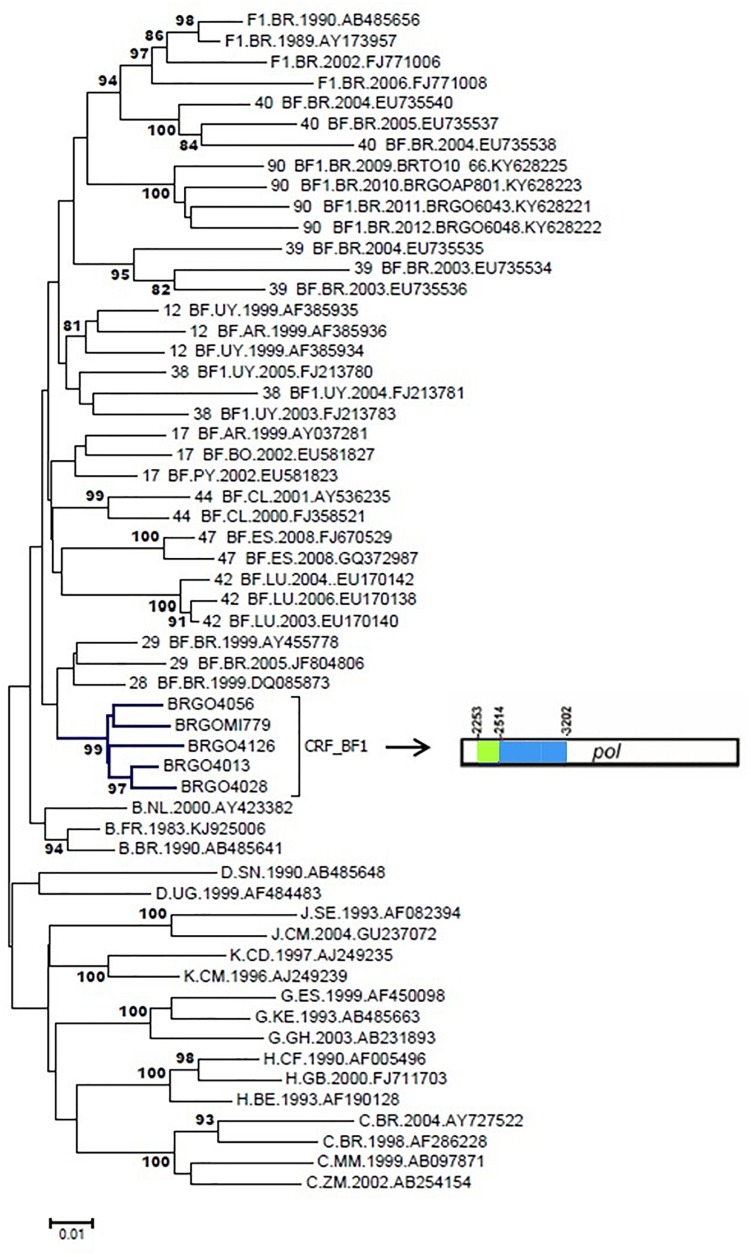
Phylogenetic analysis of five *pol* BF1 sequences with similar recombination profile. NJ method and Kimura two-parameter evolutionary model/1,000 replicate *bootstrap* values were applied. In the mosaic structure, the green color stands for subtype F1 and the blue color stands for subtype B.

In the current study five BF1 isolates from Cluster #2 (described in Reis et al., 2017) (BRGOMI779, BRGO4013, BRGO4028, BRGO4056, and BRGO4126) were further sequenced (Figure 6). Among these five BF1 isolates from Cluster #2 we were able to generate the full-length genome sequence of two viruses, the BRGO4056 strain (407–9616 bp) and the BRGO4028 strain (363–9612 bp) and the near-full-length genome sequence of BRGO4013 strain (350–7335 bp and 7750–8576 bp) relative to HXB2 genome positions.

**FIGURE 7:**
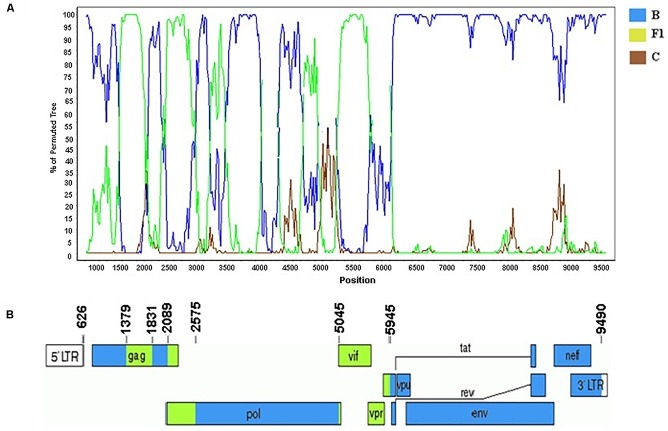
Mosaic structure of the new CRF99_BF1. **(A)** Bootscanning analysis was conducted using a window size of 300 bp and a step size of 20 bp along with reference strains of B, C, and F1 representative HIV-1M subtypes. **(B)** Genomic structure of the CRF99_BF1. Breakpoint positions according to HXB2 genome numbering system are indicated. The blue color stands for subtype B, the green color stands for subtype F1, and the brown color for subtype C. The mosaic map was generated using the Recombinant HIV-1 Drawing Tool (www.hiv.lanl.gov/content/sequence/DRAW_CRF/recom_mapper.html).

According to the standardized nomenclature by the Los Alamos National Laboratory the full and near-full sequences of these BF1 strains were designated as CRF99_BF1 (BP = 99%). The mosaic structure of the CRF99_BF1 showed the predominance of subtype B (Figure 7). The bootscanning analysis of the whole genome of the CRF99_BF1 showed six recombination breakpoints which divide the genome into seven subregions (Figure 7). According to this, the following subregions and subtypes were identified in the full-genome sequence of BRGO4056 strain: subtype B (626–1378 bp; 1831–2088 bp; 2575–5044 bp; and 5945–9601 bp) and subtype F1 (1379–1830 bp; 2089–2574 bp; and 5045–5944 bp) all of them relative to HXB2 genome positions.

The three strains that make up the CRF99_BF1 were obtained from pregnant women (17-, 25-, and 31-year-old) all of them ARV-naïve living in Goiás state and recruited from 2008 to 2009.

No other HIV-1 *pol* sequence sharing a mosaic profile similar to the sequences that make up the new CRF99_BF1 was identified in our BLAST searches. The estimated median TMRCA of the CRF99_BF1 was previously reported to be 1993, ranging from 1985 to 1998 (Reis et al., 2017).

### Characterization of Two New URFs_BF1

**FIGURE 8:**
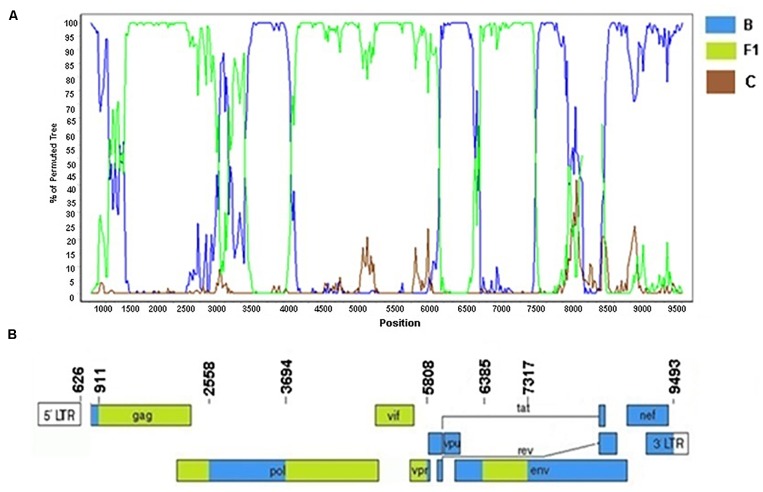
Recombination breakpoint analyses of URF_BF1/BRGOMI744. **(A)** Bootscanning analysis was conducted using a window size of 300 bp and a step size of 20 bp along with reference strains of B, C, and F1 representative HIV-1 M subtypes. **(B)** Genomic structure of URF/BRGOMI744. The mosaic map was generated using the Recombinant HIV-1 Drawing Tool (www.hiv.lanl.gov/content/sequence/DRAW_CRF/recom_mapper.html).

Among six HIV-1 BF1 sequences (BRMA94, BRMA95, BRPI96, BRMA242, BRGO4122, and BRGOMI744) that formed Cluster #1 (described in Reis et al., 2017) we were able to obtain the near-full-length sequence of the BRGOMI744 strain (411-9617 bp) from Goiás State. For the BRMA94 strain, from Maranhão State, only partial genome sequence of fragments were obtained (1028–5930 bp, 6004–7765 bp, relative to HXB2 genome positions). The bootscanning analysis of the near-full genome sequence of the BF1 strain BRGOMI744 showed a complex pattern comprising six recombination breakpoints which divide the genome into seven subregions (Figure 8). The following subtypes and subregions were identified in this genome: subtype B (626–910 bp; 2558–3693 bp; 5808–6384 bp; and 7317–9493 bp) and subtype F1 (911–2557 bp; 3694–5807 bp; and 6385–7316 bp) all of them relative to HXB2 genome positions. The partial genome fragments of the BRMA94 strain presented the same mosaic structure as described in the BRGOMI744 strain.

**FIGURE 9:**
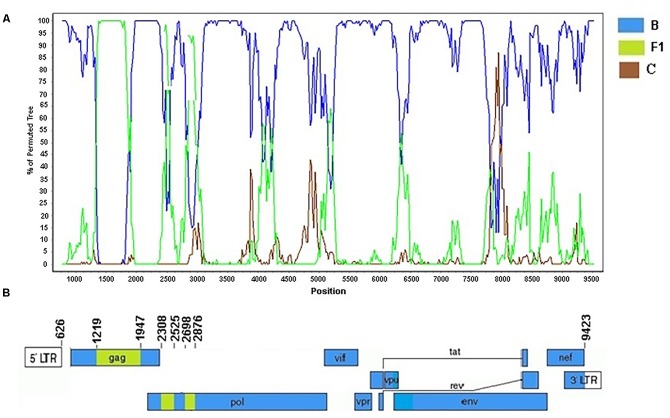
Recombination breakpoint analyses of the URF_BF1 identified in the BRMT1319 strain. **(A)** Bootscanning analysis was conducted using a window size of 300 bp and a step size of 20 bp along with reference strains of B, C, and F1 representative HIV-1 M subtypes. **(B)** Genomic structure of the URF_BF1 identified in the BRMT1319 strain. The mosaic map was generated using the Recombinant HIV-1 Drawing Tool (www.hiv.lanl.gov/content/sequence/DRAW_CRF/recom_mapper.html).

The Cluster #3 (described in Reis et al., 2017) comprised four isolates (BRGO3153, BRGO4057, BRMT1319, and BRMT2835). The full-genome sequence of the BRMT1319 strain showed that this URF_BF1 is mainly composed of subtype B. The bootscanning analysis of this genome showed six recombination breakpoints, dividing the genome into seven subregions (Figure 9). The subtypes and subregions identified in this genome were the following: subtype B (626–1218 bp; 1947–2307 bp; 2525–2697 bp; and 2876–9423 bp) and subtype F1 (1219–1946 bp; 2308–2524 bp; and 2698–2875 bp) all of them relative to HXB2 genome positions.

## Discussion

Our study with near-full and full-length genome sequences of recombinant viruses describes two new HIV-1 CRFs: the BF1C CRF81_cpx and the CRF99_BF1 that were identified among over 800 patients living in six Brazilian states. These states are located across three different geographic areas where the prevalent subtype B cocirculates with minor subtypes F1 and C. Our study also characterized two URFs BF1 in this geographic area. This is the first description of a CRF_cpx in Brazil and it is the first among 23 CRF_cpx already reported at Los Alamos HIV Sequence Database, that does not contain subtype A (http://www.hiv.lanl.gov, last accessed December 2018). So far, all CRF_cpx described worldwide presented a partial subtype A genome, either “pure” or as CRF01 (subtypes A, E) or CRF02 (subtypes A, G) (http://www.hiv.lanl.gov, last accessed December 2018). The complex CRF characterized here contains nine breakpoints and 10 subregions of subtypes B, F1, and C. Our data suggest that the CRF81_cpx originated almost 20 years ago in 1999 and that it circulates in a wide geographic area including the Central Western region, where it probably originated, and the Southern Brazilian (Paraná state) region.

In addition, our analyses provided evidence that this newly described BF1C CRF81_cpx also circulates in Italy, where the HIV/AIDS epidemic has been characterized by an increasing genetic diversity of mainly non-B subtype strains (Lai et al., 2010). A previous phylogeographic study indicated an association between the Italian and the South American HIV/AIDS epidemic suggesting that the main source of the Italian subtype C epidemic is associated with interactions between Italian heterosexual and South American homosexual males (Lai et al., 2014). In the Italian study, although patients infected with subtype C mainly referred themselves as heterosexuals, homo- or bisexual contacts were considered the source of their infection (Lai et al., 2014). Although the investigation of the sexual links involved in the origin of the CRF81_cpx was out of the scope of this study, one of patients harboring the BF1C CRF81_cpx reported himself as MSM, suggesting a possible role of this category in the origin of this mosaic CRF.

Corroborating a possible link between the Italian and the South American HIV/AIDS epidemics, in our dataset we have found a CRF60_BC-like cluster represented by strains circulating in Northeastern and Central Western Brazil. The CRF60_BC was originally identified in Southern Italy after an outbreak among 22 patients, mostly highly educated, young MSM (Monno et al., 2012; Simonetti et al., 2014). Phylogenetic analysis of full-length genome of CRF60_BC revealed the South American origin of the C subtype parental strain (Simonetti et al., 2014). Similarly to the newly described CRF81_cpx, that appears to circulate in Brazil and Italy, our findings of CRF60_BF-like sequences support the connection between the HIV/AIDS epidemic in these two countries. It has been proposed that the presence of about 400,000 South American immigrants in Italy and of a significant and unstable population of transgender sex workers (around 10,000 individuals, 60% of them from South America) strenghthen this genetic association (Lai et al., 2014). Further studies are necessary to fully evaluate the spread and the geographic area of circulation of the CRF60_BC in Brazil.

Whereas we have found evidence of the spread of CRF60_BC in Brazil, up to now only one CRF containing subtypes B and C, the CRF31_BC was described in Brazil, in the Southern region, where subtype C is a main genetic variant (Santos et al., 2006). In our study we have found a CRF31_BC-like cluster indicating the dissemination of this CRF or a variant derived from it, from Southern to Central Western and Northeastern Brazil. In this study we also characterized the new CRF99_BF1 and two new URFs BF1. Currently there are 98 HIV-1 CRFs reported globally (http://www.hiv.lanl.gov, last accessed December 2018). So far, among the 15 CRF_BF reported at the Los Alamos HIV Sequence Database, the majority was described in different geographic regions of Brazil: CRF28_BF, CRF29_BF, CRF39_BF, CRF40_BF, CRF46_BF, CRF70_BF, CRF71_BF, CRF72_BF, and the CRF90_BF1 which was recently described by our group in the Central Western region (Sanabani et al., 2006, 2010; Guimarães et al., 2008; Pessôa et al., 2014a,b; Reis et al., 2017). Several URFs BF1 and URFs BC have been described by full and near-full-length genome sequencing studies in Brazil and this approach is necessary to define the real contribution of these mosaic forms in the Brazilian epidemic (Barreto et al., 2006; Sanabani et al., 2006, 2010; Passaes et al., 2009; Pessôa et al., 2016; Marques et al., 2018).

## Conclusion

The description of the first CRF_cpx composed of subtypes B, F1, and C identified in Brazil shows that more complex HIV-1 recombinant variants are circulating in the country and also in Italy. Additionally the description of the new CRF99_BF1 and of two new URFs BF1 confirms the wide generation and spread of BF1 intersubtype recombinants in the Brazilian HIV/AIDS epidemic. Our results reinforce the need to expand HIV-1 full-length genomic studies in Brazil and worldwide in order to estimate the overall proportion of intersubtype recombinants in the HIV/AIDS epidemic.

## Ethics Statement

This study was carried out in accordance with the recommendations of “Resolução 196/96 da CONEP/MS, Comite de Ética e pesquisa do Hospital de Doenças Tropicais/GO; CEPMHA/HC/UFG no186; 073/05, Comite de Ética em Pesquisa Médica Humana e Animal; No435/CEP-HUJM/07, Comite de Ética em Pesquisa do Hospital Universitário Júlio Miller; CEP-UESPI 022/2011, Comite de Ética em pesquisa da Universidade Estadual do Piauî with written informed consent from all subjects. All subjects gave written informed consent in accordance with the Declaration of Helsinki. The protocol was approved by the “Comite de Ética e pesquisa do Hospital de Doenças Tropicais, Comite de Ética em Pesquisa Médica Humana e Animal, Comite de Ética em Pesquisa do Hospital Universitário Júlio Miller, Comite de Ética em pesquisa da Universidade Estadual do Piauî.

## Author Contributions

MS conceived, designed and received funding for the study. MS and MR collected and analyzed the epidemiological data. MR, MG and GB performed the phylogenetic analyses. GB performed the evolutionary analyses. MR performed all figures and tables. MS and MR wrote the manuscript. All authors discussed and reviewed the manuscript.

## Conflict of Interest Statement

The authors declare that the research was conducted in the absence of any commercial or financial relationships that could be construed as a potential conflict of interest.
