# Supplementary material for: Identification of New HIV-1 Circulating Recombinant Forms CRF81_cpx and CRF99_BF1 in Central Western Brazil and of Unique BF1 Recombinant Forms
Source: Front Microbiol. 2019 Feb 11;10:97. doi: 10.3389/fmicb.2019.00097 (PMC6378278; doi:10.3389/fmicb.2019.00097)
Supplement: Supplementary file 1 [file Table_1.DOCX]

**Supplementary Table 1. Summary of HIV-1 subtypes identified in *pol* sequences from previous studies among patients from six Brazilian States: Goiás/ GO, Mato Grosso/ MT, Mato Grosso do Sul/MS, Tocantins/TO, Piauí/PI and Maranhão/MA**

| **Reference** | **Sample Collection (Year)** | **State/Region** | ***pol* sequences (n)** | **HIV-1 Subtypes** | | | | | | |
| --- | --- | --- | --- | --- | --- | --- | --- | --- | --- | --- |
|  |  |  |  | **B** | **C** | **F1** | **BF1** | **BC** | **F1CB** | **CF1** |
| Cardoso et al 2009 | 2007-2008 | GO/Central West | 97 | 80 | 3 | 6 | 7 | - | 1 | - |
| Cardoso & Stefani 2009 | 2007-2008 | GO/Central West | 48 | 38 | 1 | 2 | 7 | - | - | - |
| Cardoso et al 2010 | 2003 | GO/Central West | 77 | 56 | 3 | 5 | 13 | - | - | - |
| Cardoso et al 2011 | 2008-2009 | GO/Central West | 7 | 6 | - | - | 1 | - | - | - |
| Cardoso et al 2011 | 2008-2009 | MS/Central West | 20 | 10 | 3 | - | 7 | - | - | - |
| Ferreira et al 2011 | 2008-2009 | MT/Central West | 92 | 67 | 5 | 3 | 11 | 4 | 2 | - |
| Carvalho et al 2011 | 2008-2009 | TO/North | 52 | 42 | 3 | 1 | 4 | - | - | 2 |
| da Silveira et al 2012 | 2008-2010 | MS/Central West | 49 | 32 | 5 | 4 | 4 | 4 | - | - |
| Alcântara et al 2012 | 2008-2010 | GO/Central West | 83 | 51 | 11 | 4 | 15 | 2 | - | - |
| da Costa et al 2013 | 2010-2011 | GO/Central West | 18 | 14 | 3 | 1 | - | - | - | - |
| Moura et al 2015a | 2012-2013 | MA/Northeast | 106 | 91 | 3 | 2 | 8 | 3 | - | - |
| Moura et al 2015b | 2011-2012 | PI/Northeast | 89 | 77 | 1 | 1 | 4 | 6 | - | - |
| Lima et al 2016b | 2010-2013 | GO/Central West | 90 | 66 | 12 | 6 | 6 | - | - | - |
| Total |  |  | 828 | 629 | 53 | 35 | 87 | 19 | 3 | 2 |
